# Tuning the Covering on Gold Surfaces by Grafting Amino-Aryl Films Functionalized with Fe(II) Phthalocyanine: Performance on the Electrocatalysis of Oxygen Reduction

**DOI:** 10.3390/molecules26061631

**Published:** 2021-03-15

**Authors:** Camila F. Olguín, Nicolás Agurto, Carlos P. Silva, Carolina P. Candia, Mireya Santander-Nelli, Juan Oyarzo, Alejandra Gómez, Juan F. Silva, Jorge Pavez

**Affiliations:** 1 Universidad de Santiago de Chile (USACH), Faculty of Chemistry and Biology, Department of Chemistry of the Materials, Soft Matter Research and Technology Center, SMAT-C. Av. Libertador B. O’Higgins 3363, Box 40, Correo 33, Santiago 9170022, Chile; camila.olguinc@usach.cl (C.F.O.); nicolas.agurto@usach.cl (N.A.); carolina.candiac@usach.cl (C.P.C.); mireya.santander@usach.cl (M.S.-N.); alejandra.gomez@usach.cl (A.G.); juan.silva.r@usach.cl (J.F.S.); 2 Instituto de Química, Pontificia Universidad Católica de Valparaíso, Valparaíso 2374631, Chile; juan.oyarzo.m@pucv.cl

**Keywords:** grafting covering control, aryl diazonium salt, Fe(II) Phthalocyanine, oxygen reduction reaction

## Abstract

Current selective modification methods, coupled with functionalization through organic or inorganic molecules, are crucial for designing and constructing custom-made molecular materials that act as electroactive interfaces. A versatile method for derivatizing surfaces is through an aryl diazonium salt reduction reaction (DSRR). A prominent feature of this strategy is that it can be carried out on various materials. Using the DSRR, we modified gold surface electrodes with 4-aminebenzene from 4-nitrobenzenediazonium tetrafluoroborate (NBTF), regulating the deposited mass of the aryl film to achieve covering control on the electrode surface. We got different degrees of covering: monolayer, intermediate, and multilayer. Afterwards, the ArNO_2_ end groups were electrochemically reduced to ArNH_2_ and functionalized with Fe(II)-Phthalocyanine to study the catalytic performance for the oxygen reduction reaction (ORR). The thickness of the electrode covering determines its response in front of ORR. Interestingly, the experimental results showed that an intermediate covering film presents a better electrocatalytic response for ORR, driving the reaction by a four-electron pathway.

## 1. Introduction

There is considerable interest in building molecular platforms based on the selective modification of electro-active surfaces to improve electrodic materials’ performance. Modifying surfaces through grafting and electrografting via diazonium salts has arisen as an alternative approach. This methodology is considered better than other alternatives since the grafting conditions can be easily controlled and adapted to many substrate types, including carbon, metals, and oxides [1,2,3,4,5,6]. However, this strategy has a drawback due to the high reactivity of aryl radicals generated at the interfacial zone. These radical species react with the previously grafted aryl group on the electrode surfaces, generating a massive deposition of polyaryl layers in situ; therefore, there is no control of the thickness and organization [7,8]. Many efforts have been made to prevent the formation of multilayers from gaining greater control of the interface. Among these, the following strategies stand out: including a bulky group of aryl diazonium derivatives (steric hindrance) [9], employing ionic liquids to control the diffusion [10], and using the selective redox cross-inhibitor. This final strategy arises from a series of works reported by Breton and co-workers [11,12,13]. Taking into account, as is well known, that the electrografting mechanism via DSRR is driven by a highly reactive aryl radical, they started using a radical scavenger (RS) to control the grafting mechanism of a nitro-aryldiazonium salt on a GC electrode, and achieved the deposition of monolayers of grafted aryl film. The same group, working with a series of aryl diazonium salts substituted with an electron-withdrawing group in *para* position, found that a nonradical secondary mechanism on the layer growth would take place depending on the activating or deactivating character of the substituent, and the control exercised by the RS depends on this property [14]. Later, the same authors proposed that the effective control of the RS on film growth is due to a redox cross-reaction that takes place in the interfacial zone between the diazonium cation and the reduced form of RS, and the difference between their formal potentials allows for fine control by cyclic voltammetry on the final covering on the electrode surface [11]. Although these approaches have always tried to promote monolayer grafting or much thinner films, especially for nanotechnology devices, little attention has been given to the study of intermediate or multilayer coatings. In this work we used the redox cross-reaction approach to obtain different relative thicknesses of grafted aryl films.

For electrocatalytic studies on modified electrodes with electroactive coatings, it is essential to consider how the relative thickness film affects the interfacial charge transfer process and its kinetics and mechanism. We assess this effect through the oxygen reduction reaction (ORR), a very sensitive reaction that takes into account the electronic and structural conditions of the interface. The ORR is a critical reaction involved in fuel cell devices for energy conversion [15,16]. It is well known that the main drawback of ORR is its slow kinetics, and the best catalysts used for this reaction are platinum-based cathodes, but the high cost of Pt has limited the widespread use of this technology. Today, nonprecious metal catalysts have become an excellent alternative to replace platinum. Among them are the MN4 macrocycle catalysts like Fe(II) and Co(II) phthalocyanines [17,18,19,20,21] or porphyrins [22,23,24].

**Scheme 1:**
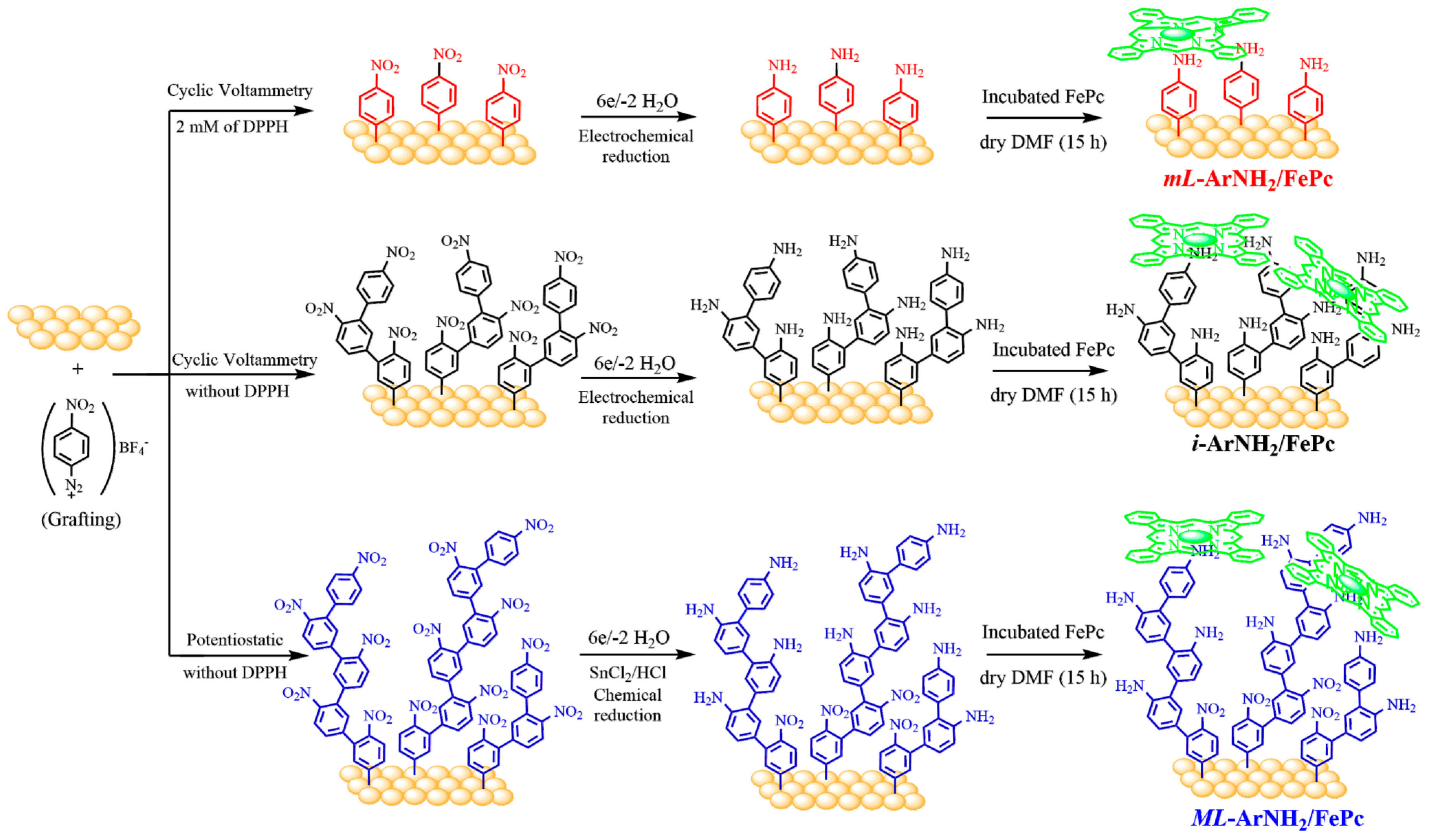
Diagram for the general procedure of electrografting on the electrode surfaces with details of the subsequent modifications until the incorporation of the FePc catalytic complex.

In this work we provide insight into the effect of the relative covering degrees of grafted nitro-aryl films; monolayer coverage, *mL*-ArNO_2_; and two massive surface coverage (intermediate, *i*-ArNO_2_; and multilayer, *ML*-ArNO_2_) on the electrocatalytic activity for ORR. We prepare modified gold electrodes through diazonium salts (4-nitrobenzenediazonium) electroreduction technique. The grafted nitro-aryl films are electrochemically reduced and functionalized with Fe(II) Phthalocyanine (FePc), obtaining the study systems *mL*-ArNH_2_/FePc, *i*-ArNH_2_/FePc, and *ML*-ArNH_2_/FePc. Scheme 1 illustrates the general procedure of electrografting on the gold electrode surfaces and the subsequent functionalization with the FePc complex. The aim of incorporating the FePc catalyst complex is to assess the covering of the electrode surface to optimize the interfacial electron transfer in the electrocatalytic process of ORR.

## 2. Results and Discussion

### 2.1. Electrografting of Gold Electrode whit 4-Nitrophenyl Diazonium

**Figure 1:**
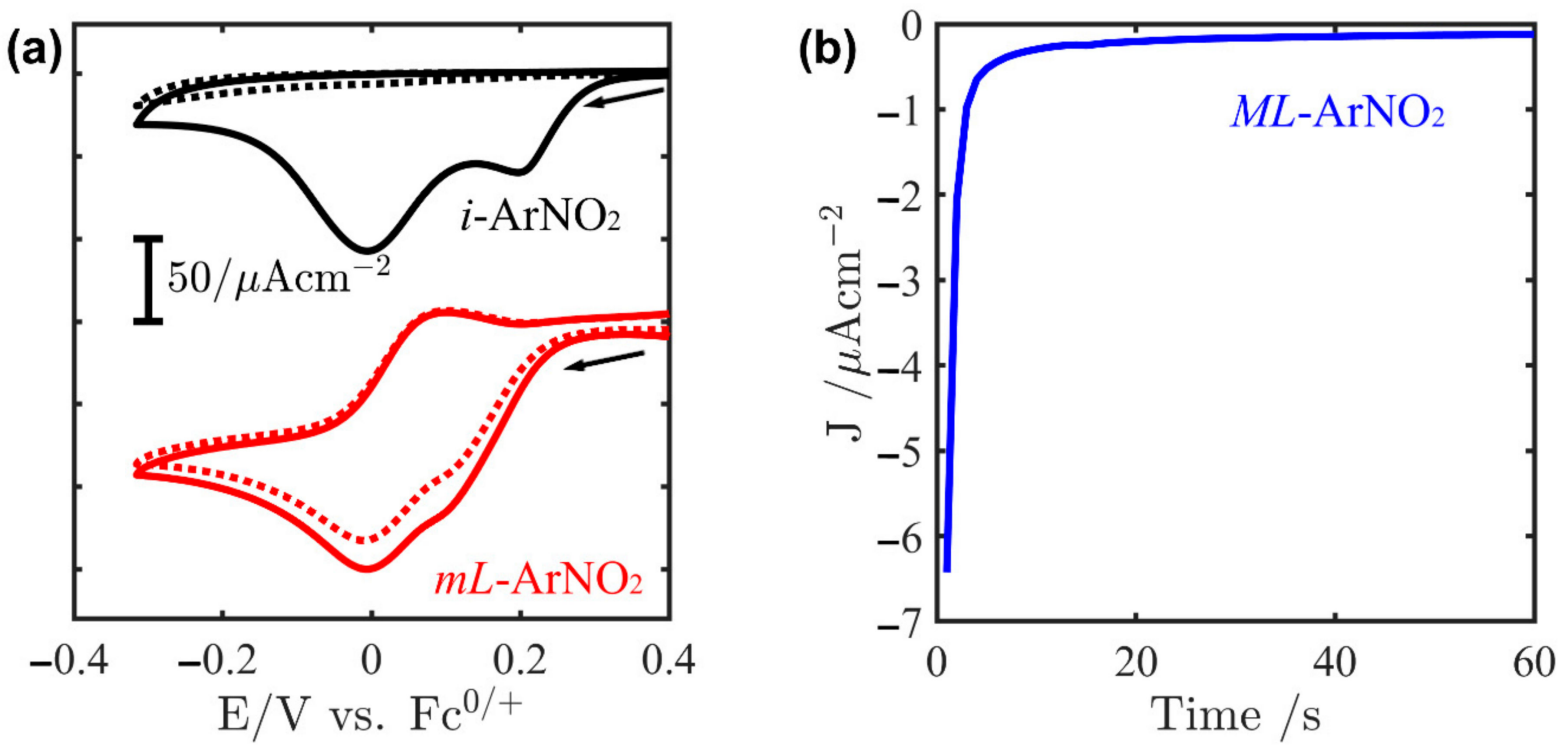
(**a**) First (solid line) and second (dotted line) CV modification for *i*-ArNO_2_ (black line), *mL*-ArNO_2_ (red line); (**b**) time-current curve to *ML*-ArNO_2_ (blue line) recorded in ACN 0.1M TBAP on a gold electrode.

Figure 1 shows the electrografting by cyclic voltammetry (CV) for the intermediate (*i*-ArNO_2_) and monolayer (*mL*-ArNO_2_) covering systems, and the electrografting at fixed potential for the *ML*-ArNO_2_ covering system. In Figure 1a, the typical voltammogram recorded for the *i*-ArNO_2_ system, in a black line, shows two characteristic cathodic processes for electrochemical reduction of 4-nitrophenyl on gold (peaks at 0.2 y 0.0 V (vs. Fc^1+/0^)) [25]. Benedetto et al. [26] attributed these processes to the reduction of aryldiazonium derivative on different polycrystalline gold faces (predominantly Au(111) and Au(100)). The flattening of the voltametric response observed in the second cycle of the voltammogram is due to the possible blockage of the electrode surface by the formation of a compact organic layer (*i*-ArNO_2_) by the grafting process [6]. The lower voltammogram in Figure 1a, which represents the monolayer electrografting (*mL*-ArNO_2_) covering systems, was recorded with two DPPH equivalents added to the electrolyte solution. It shows an additional reduction process at 0.00 V, which corresponds to the anion radical formation from the radical scavenger (DPPH^−^), overlapping with the diazonium reduction process [12]. Nevertheless, when the second cycle of the voltammogram is finished, a very weak passivation of the surfaces is observed, which is consistent with a monolayer thin film covering the electrode surface. On the other hand, according to other workgroup studies, when applying a fixed potential, multilayers of aryl groups are formed on the electrode surface [27,28,29]. Thus, chronoamperometric experiments were carried out by applying a constant potential for 1 min, using twice the concentration of diazonium (2 mM NBTF) (Figure 1b). The chronoamperogram showed a decrease in current (until 5 s), indicating an increase in the surface coverage by the nitrophenyl group formed by aryl radicals. Afterward, the electrode charge (1.75 × 10^−5^ C) was obtained through a current-time graph, which corresponded to a 3.6 × 10^−9^ mol cm^−2^ surface concentration, a value that, as expected, is associated with a multilayer covering of nitrophenyl grafted layers (*ML*-ArNO_2_) [30].

**Figure 2:**
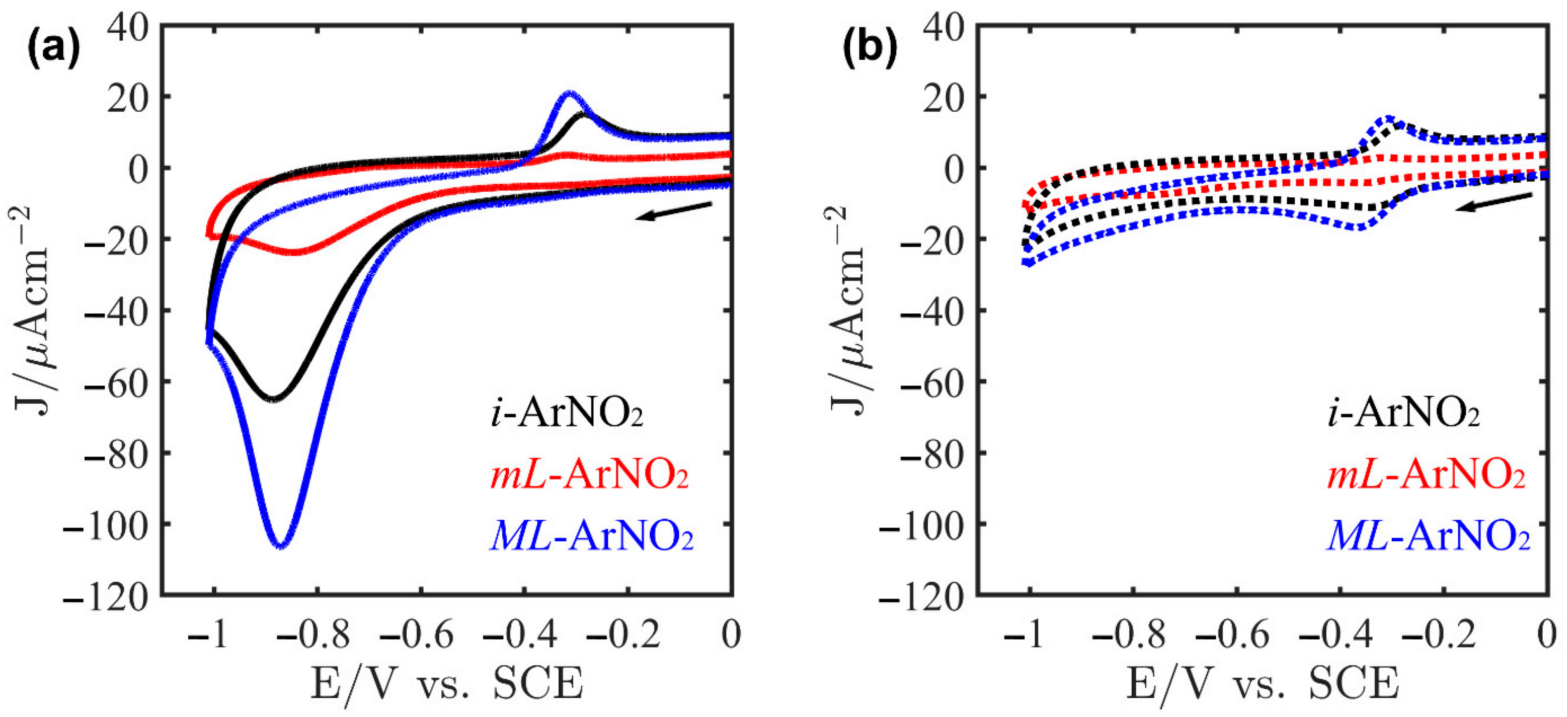
Cyclic voltametric response for the electrochemical reduction of surface-bound nitrophenyl groups; *i*-ArNO_2_ (black), *mL*-ArNO_2_ (red), and *ML*-ArNO_2_ (blue) in N_2_-saturated Britton–Robinson buffer (pH: 11) at 50 mV s^−1^. (**a**) First scan (solid line); and (**b**) second scan (dotted line).

After grafting, an electrochemical reduction of surface-bound nitrophenyl groups was performed. This procedure allows for generating Ar–NH_2_ terminal function on the grafts, which will act as the fifth axial ligand for coordination of the iron (II) phthalocyanine (FePc) catalyst complex to the surface of the modified electrode [24,31]. There have been several studies on the pH conditions for the electrochemical reduction of nitrophenyl groups on various surfaces [12,28,32]. Under acidic conditions, the nitro groups (ArNO_2_) are partially reduced to amino groups (ArNH_2_/6ē) and phenylhydroxylamine (ArNHOH/4ē). However, the most suitable way to calculate the surface covering is to carry out the electrochemical reduction in an alkaline medium, with which it is possible to determine the surface covering associated with the ArNO_2_/ArNH_2_ (6ē) process [12]. Figure 2 shows the electrochemical response to the reduction of surface-bound nitrophenyl groups in a Britton–Robinson pH 11 buffer solution. In the first scan (Figure 2a), for all systems, the irreversible reduction of the nitro group was seen at −0.88 V, attributing to amine (Ar-NH_2_) and hydroxylamine formation (ArNHOH) [25,32]. On the anodic sweep at −0.31 V, an oxidation process of ArNHOH (produced during the cathodic sweep) to Ar–NO was observed. In the second scan (Figure 2b), the reversible process observed at −0.34 V is associated to Ar–NO/Ar–NHOH redox couple. Remarkably, no significant contribution to the cathodic process associated to Ar–NO_2_ reduction (~−0.88 V) was found on the second scan, showing the total reduction of the accessible Ar–NO_2_ groups in the grafted layers, as reported by previous works about grafting on carbon and gold electrodes [6,25,30].

In all systems, the relative covering degrees of films (the surface concentration in mol cm^−2^) were estimated, considering the charge by integrating the cyclic voltammogram area of Figure 2. Table 1 shows that both the potentiodynamic modification (without DPPH) and the modification by fixed potential (potentiostatic) have a statistically comparable thickness, i.e., there is no significant difference in their coverings. Despite this, the ML-ArNO_2_ system gave a value of superficial concentration that, as we pointed out above, correlates with multilayer-like grafting. This result could be explained considering a possible covering structure based on multilayer grafting arranged in a three-dimensional form, where not all end-NO_2_ groups present would be spatially available for the electrochemical reduction in this multilayer system (*ML*-ArNO_2_). Consequently, in the multilayer system, we could not correlate the surface concentrations value with the electrodes charge, since the response corresponds just to the available electroactive NO_2_ groups and not to the whole species grafted on the electrode surface [33]. In consequence, for the ML-ArNO_2_ system a full reduction was achieved by chemical reduction in the presence of SnCl_2_ to ensure the entire reduction of the nitro groups present in the internal layers of the system [13]. The system obtained by using DPPH (mL-ArNO_2_/mL-ArNH_2_), presented a lower surface thickness, where the estimated coverage was 1.91 × 10^−10^ mol cm^−2^. Mananteu et al. reported that using glassy carbon (GC) as a working electrode and modifying the surface under the same conditions used in our work presented a thickness of 6.4 × 10^−10^ mol cm^−2^ (in the presence of DPPH) that would correspond to a grafted monolayer [12]. The difference between these coating values for monolayers could be attributed to the nature and roughness of the work electrodes used (gold vs. GC).

### 2.2. Electric Properties of Grafted Films; Electrochemical Impedance Spectroscopy

**Figure 3:**
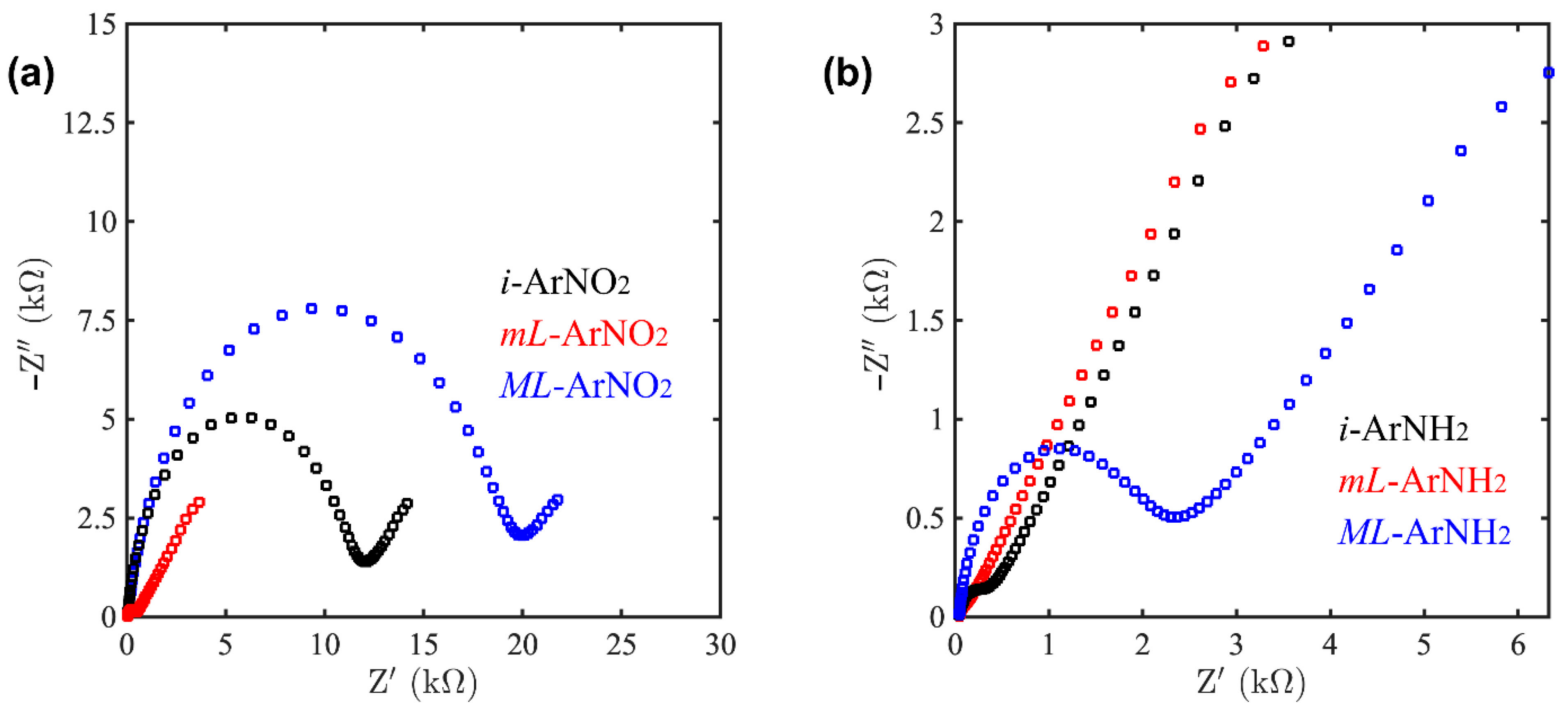
Impedance spectra (Nyquist plots) obtained from the modification (**a**) Au/*i*-ArNO_2_ (black), Au/*mL*-ArNO_2_ (red) and Au/*ML*-ArNO_2_ (blue); (**b**) Au/*i*-ArNH_2_ (black), Au/*mL*-ArNH_2_ (red) and Au/*ML*-ArNH_2_ (blue); polycrystalline gold electrode with 2.5 mM Fe(CN)_6_^3−^/^4−^ each, and 0.5 M KNO_3_ support electrolyte.

Figure 3 shows the impedance spectra for organic films through Nyquist plots. The impedance response of these grafted films strongly depends on the thicknesses of covering films and the nature of the terminal function (end group) on the grafts. For both grafted film groups (Ar–NO_2_ and Ar–NH_2_), the impedance spectra show the characteristic response of semicircles at high frequencies that fits with a Randles circuit, corresponding to a combination of the charge transfer resistance (R_ct_), at the film/electrolyte interface, with double-layer capacitance, and for the appearance of a marked diffusion-controlled region at middle and low frequencies in the case of the Ar–NH_2_ grafted films. The diameter of semicircles is indicative of the charge transfer resistance. In all cases, it decreases with the thickness of the covering film, no matter the nature of the end group on the grafts. From the diameter of semicircles of the thicker films (grafting without DPPH), intermediate (*i*) and multilayer (*ML*), the charge transfer resistance of the electrochemical process is estimated to be controlled by the mass-transfer resistance, and by charge-transfer (kinetic) resistance in the case of the monolayer (*mL*) covering films.

Comparing the impedance spectra of Ar–NO_2_ (Figure 3a) and Ar–NH_2_ (Figure 3b) shows that the graft end groups nature determines the impedance response. The impedance spectra and data from Table 2 clearly show that, after the electrochemical reduction of NO_2_ end groups, the covering films become more electroactive. These Ar–NH_2_ grafts, especially the thinner ones, show R_ct_ values three times lower than the Ar–NO_2_ grafts and also show higher capacitance values, which indicates a more charged interface. Another critical feature of reduced thinner grafts (intermediate and monolayer) is the appearance of a noticeable Warburg line at moderately high and high frequency values, which shows essential diffusion-controlled processes that contribute to the impedance of the system. This behavior is evidence that the electrochemical reduction of NO_2_ end groups of grafted films becomes more effective on thin (intermediate and monolayer) films than thick films. The R_ct_ values for these systems are comparable to what was reported concerning the monolayer, intermediate, and thick organic films on a glassy carbon and gold surface. The increase in the charge transfer resistance value is a direct consequence of the gradually increasing thickness [26]. This reduces the capacity of the redox probe to access the gold surface due to the increasing thickness of the organic film acting as a barrier to the electron transfer [11]. In this context, the *ML*-ArNO_2_ system presented great magnitude charge-transfer resistance, even after being reduced. These results provide additional evidence that a considerable fraction of nitro groups in the *ML*-ArNO_2_ system would not be suitable for full electroreduction (see Section 2.1).

### 2.3. Surface Morphology of Amino-Aryl Grafting

**Figure 4:**
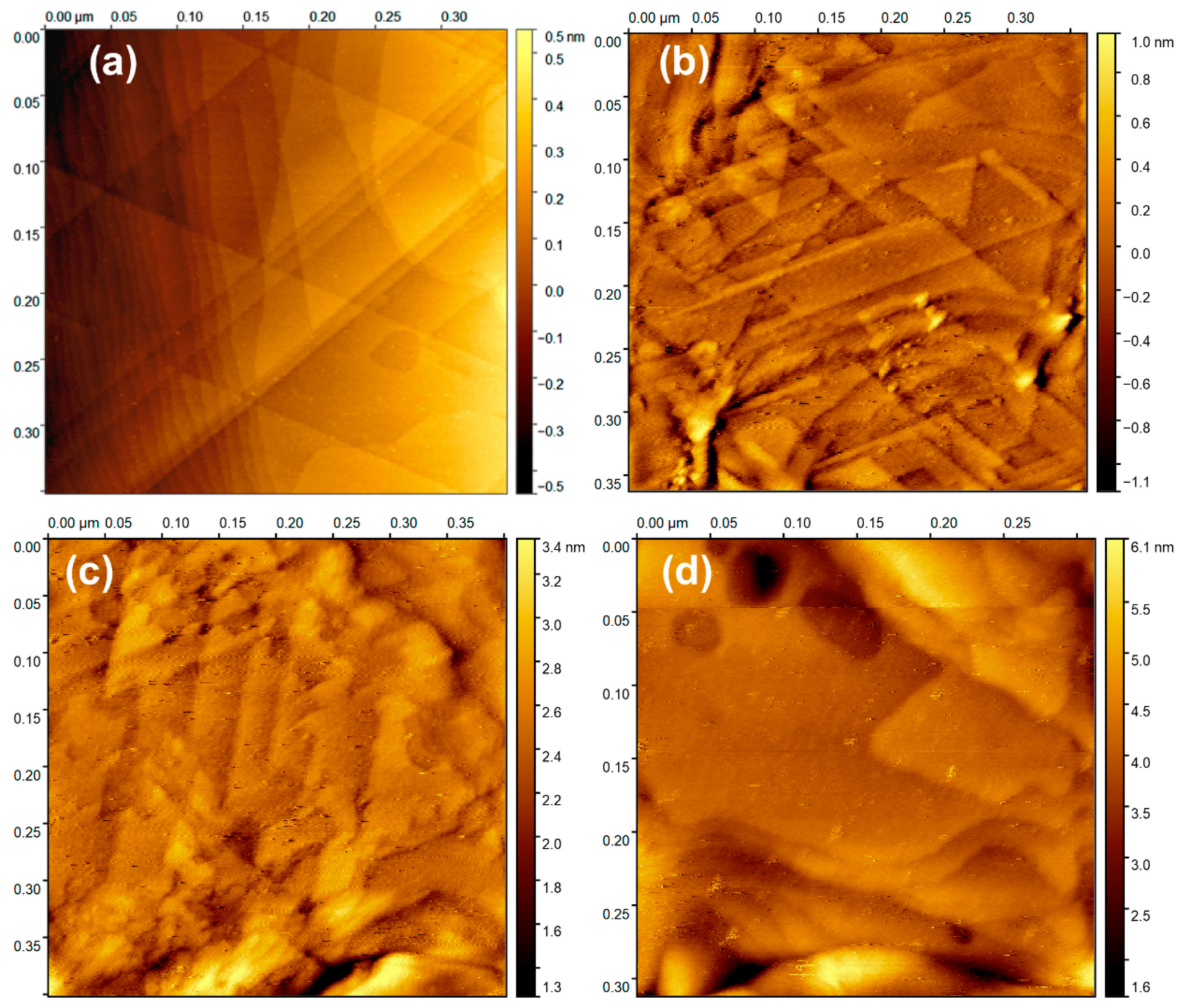
STM images of air conditions, collected in constant-current mode, of modified surface electrodes: (**a**) bare gold electrode; (**b**) Au/*mL*-ArNH_2_; (**c**) Au/*i*-ArNH_2_; and (**d**) Au/*ML*-ArNH_2_.

The surface morphology of a modified electrode with amino-aryl grafting was studied by scanning tunneling microscopy (STM). Figure 4 shows STM images of the surface morphology of the Au/amino-aryl modified electrode systems generated by the methods described above. The image in Figure 4a corresponds to the bare electrode surface. It is observed that its morphology is characterized by smooth terraces and a relatively high density of atomic steps, as revealed by the low-resolution STM image. From the images in Figure 4c,d, it can be seen how the morphology of the surface of the modified electrode evolves as the covering of the deposited graft increases. On the gold surface modified with a monolayer (mL) of amino-aryl grafting (Figure 4b), it is possible to observe that a smooth morphology of terraces and atomic steps’ border is maintained within a significant range. This scenario changes drastically as the deposited graft coating increases, as shown in the images in Figure 4b,c representing the intermediate (i) and multilayer (ML) coatings. It is clearly observed how the surface evolves from a smooth to a rough morphology due to more massive grafting, being located on both the terraces and the border of the steps, especially in the thickest film (ML), where the grafting was produced without DPPH, obtaining a very rough film in comparative terms.

### 2.4. Electrocatalysis of ORR on Au/ArNH_2_/FePc Systems

**Figure 5:**
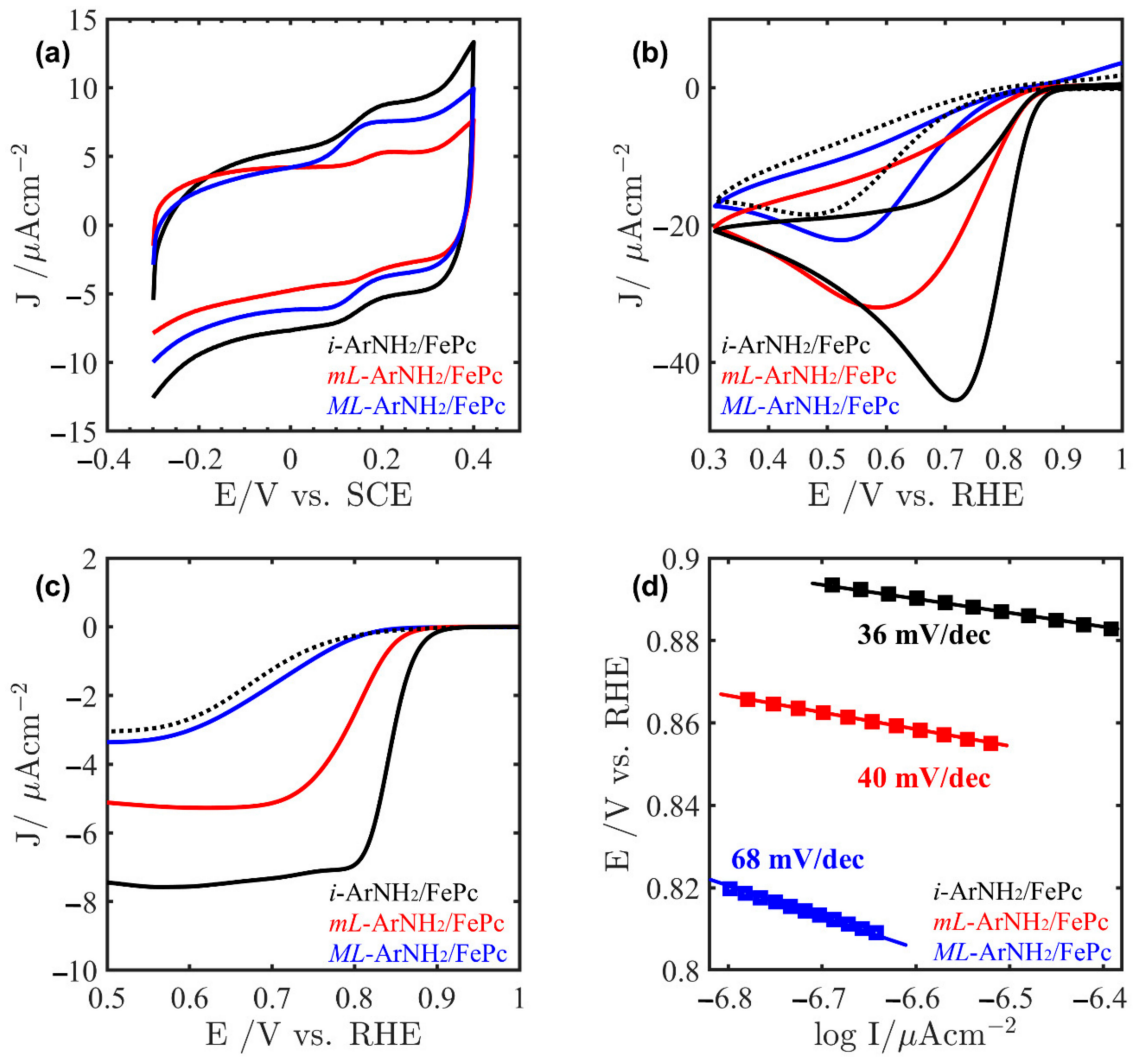
(**a**) Electrochemical characterization by cyclic voltammetry of modified electrodes: Au/*i*-ArNH_2_/FePc (black), Au/*mL*-ArNH_2_/FePc (red), and Au/*ML*-ArNH_2_/FePc (blue) in N_2_-saturated Britton–Robinson buffer (pH: 3) at 50 mV s^−1^; (**b**) electroreduction of O_2_ on bare Au electrode (dotted line) and modified electrodes in 0.1 M NaOH at 50 mV s^−1^ in saturated O_2_; (**c**) polarization curves by linear sweep voltammetry of modified electrode: *i*-ArNH_2_/FePc (black), *mL*-ArNH_2_/FePc (red) and *ML*-ArNH_2_/FePc (blue) in 0.1 M NaOH at 1mV s^−1^ in saturated O_2_; (**d**) Tafel plots.

As we pointed out above, the grafted nitro-aryl films reduced to amino-aryl were functionalized with the FePc catalyst complex to assess the electrode surface covering to optimize the interfacial electron transfer in the electrocatalytic process of ORR. Figure 5 shows the electrochemical characterization of grafted electrodes and its performance in the electrocatalysis of ORR. Figure 5a shows the electrochemical characterization of grafted electrodes functionalized with FePc in an N_2_-saturated Britton–Robinson buffer solution (pH: 3) at 50 mV s^−1^.

The voltametric profiles presents a reversible redox process around 0.15 V vs. SCE, which is attributed to the Fe(III/II) transition [21,24,34]. The presence of this characteristic redox pair demonstrates the incorporation of the macrocycle in the electrode systems and the role of the amino group as an appropriate anchoring site for the FePc catalyst on the grafted electrodes. Considering that more anchoring sites (N donor) are available and accessible for the coordination of the catalytic complex, there will be a more significant number of active sites (anchored FePc) for ORR on the electrode surface. We can estimate the number of active redox FePc sites from the electrical charge under the redox peak of voltammograms in Figure 5a and located at ca. 0.15 V, assuming that one electron per iron center is transferred. The total amounts obtained of apparent electroactive sites are 5.08 × 10^−12^, 2.87 × 10^−11^, and 1.09 × 10^−11^ mol cm^−2^, for *mL*-ArNH_2_/FePc, *i*-ArNH_2_/FePc, and *ML*-ArNH_2_/FePc, respectively. The lower value is in good agreement with the monolayer formation of FePc coverage on the amine-aryl graft [24,34]. No substantial increase in the faradaic currents is observed by increasing the covering in the thicker films (grafting without DPPH). The number of detected FePc active sites in thicker films (intermediate, *i*, and multilayer, *ML*) agrees with what was stated in previous sections regarding the whole NO_2_ species grafted on the electrode surface not becoming electrochemically reduced to NH_2_ species. Furthermore, the thickest (ML-ArNH_2_) film shows a lower peak current of the Fe(III/II) process than the intermediate covering. Therefore, the rather small variation in the number of FePc species anchored in thicker films confirmed that a significant number of active sites would not be achieved simply by increasing the degree of covering; the chemical and structural nature of the covering is decisive, as in this case.

Figure 5b–d shows the results of the electrocatalytic process of ORR. Figure 5b compares the cyclic voltammetry response of the gold electrode in the presence of oxygen before and after modification with amino-aryl grafts functionalized with the FePc catalyst complex. The current intensity for all systems varied linearly with the square root of the potential scan rate, which corroborates that the ORR is under mass transport control. The dashed line shows the response of the bare gold electrode. ORR on gold is known to proceed only via two electrons to give peroxide [35,36], so the voltametric response corresponds to a total transfer of two electrons. On the other hand, it is important to note that modified electrodes with FePc in alkaline media catalyze the ORR preferentially via the four-electron pathway to give water, as shown in the literature [17,19,20,34,37]. Therefore, the enhancement of electron transfers kinetics (catalytic currents) due to the presence of anchored FePc is through the effect on the general mechanism of the reaction.

The electrocatalytic response of the thickest functionalized film is very similar to that obtained on bare gold. This result is surprising but consistent with what was stated in the previous sections regarding the availability of possible active sites in the larger covering. Although by cyclic voltammetry the detected amount of FePc species (Figure 5a) anchored in thickest films is very similar to the intermediate (i) and one order of magnitude higher than the monolayer one, the result clearly shows that these FePc species as an active site for ORR are mostly unavailable for the electrocatalytic process. As reported in the literature, due to the absence of radical scavengers (like DPPH) during the electrografting process, the radical species react with the previously grafted aryl group on the electrode surfaces, generating the deposition of polyaryl layers in situ. This, like the polyaryl network, is arranged in a three-dimensional form at the interfacial zone [7,8]. The network could prevent the free diffusion of O_2_ towards the inner layers of this three-dimensional network, thus limiting the necessary interaction of oxygen molecules with the FePc active sites. On the other hand, the results and analysis of electrochemical impedance (Section 2.2) showed that the charge transfer resistance of the thickest film is almost twice that of the thinner ones.

A pronounced increase in electrocatalytic activity was achieved after modifying the gold electrode surface with the monolayer (mL) and intermediate (i) ArNH_2_/FePc systems. The electrocatalytic process for ORR in these functionalized films has remarkably improved thermodynamically and kinetically, concerning the bare gold electrode and the one modified with the thicker film. As was observed, the Au/ArNH_2_/FePc system with intermediate covering presented the highest activity for O_2_ reduction as compared to all of the other configurations examined. In this system, the catalytic current was slightly more than double that recorded in bare gold, showing that the overall ORR mechanism preferentially proceeds via four electrons. In addition, a shift to more positive potentials for onset ORR was observed, so the intermediate Au/ArNH_2_/FePc configuration decreased the overpotential of the ORR by about 0.095 V.

The electrocatalytic response for ORR obtained with the monolayer covering of Au/ArNH_2_/FePc also shows a remarkable improvement concerning the bare gold electrode and the one modified with the thicker film. However, despite this significant improvement, it was observed that the catalytic current was not doubled compared to bare gold, as would be expected due to the presence of FePc, which promotes ORR via four electrons. Considering that the ArNH_2_ monolayer is probably not very compact, there could be free Au sites on the electrode surface where ORR can take place. Then, on the monolayer Au/ArNH_2_/FePc configuration, the ORR probably proceeds through two parallel mechanisms: by two electrons to give peroxide and by four electrons to produce water. So, in terms of both the number of electrons per O_2_ molecule transferred and the decrease in the overpotential of the reaction, intermediate and monolayer covering films present a better electrocatalytic response for ORR. These two configurations of covering could provide better conditions from an electronic and a structural point of view to optimize the interaction between the O_2_ molecule and the FePc active sites, to promote the ORR via four electrons. Assuming the rate-determining step for ORR is the transfer of one electron to the dioxygen molecule [19,22], the chance for an adequate interaction to take place between the oxygen molecule and the Fe center in FePc is decisive for the ORR mechanism. In intermediate and monolayer covering films, there are accessible and structurally adequate larger and delocalized π systems from aryl groups that probably decrease the reorganizational energy of the process, decreasing the activation energy of ORR. Furthermore, axial ligation to the Fe center by grafting via a nitrogen atom of the NH_2_ group could also favor the interaction of O_2_ with the Fe center. On the other hand, new bioinspired systems (like heme systems) for ORR have shown that incorporating axial ligands (amine, pyridine, and pyrrole type) into the MN4 complex has improved the electrocatalytic response in ORR [15,16,19,22]. It is known that, in heme systems, the axial ligation can affect the orientation of the O_2_ molecule on the Fe center.

The orientation of the O_2_ molecule with which it is adsorbed on the metal center of the MN4 complex determines its reduction mechanism [33], where for FePc the side-on configuration (on two Fe centers) promotes the reduction of O_2_ via four electrons, and the end-on configuration (to one Fe center) promotes it via two electrons. In this way, Kasai et al. reported that, in the O_2_–Fe interaction in the structure of the heme complex, the axial ligand by electronic effects can affect the orientation of O_2_ on the metal center [38], favoring the side-on interaction, since it would decrease the energy of the transition state of this configuration, facilitating the breaking of the O–O bond and with the transfer of four electrons.

Figure 5c shows the linear sweep voltammetry response in the presence of oxygen of the bare gold electrode modified with amino-aryl grafted functionalized with the FePc catalyst complex. These low-scan polarization curves provide kinetic and mechanistic information on the ORR process and, from this data, we obtained the Tafel slopes. These parameters, shown in Figure 5d, indicate a good linear correlation between Log I and potential. The Tafel slope values clearly show that the thickness of the covering affects the catalytic mechanism of ORR. The slopes obtained for the intermediate and monolayer coverings are −36 and −40 mV/dec, respectively, suggesting that the rate-determining step of ORR involves the transfer of one electron from the Fe center to O_2_ molecule, right after, or concomitant with, the O_2_ adsorption on the Fe active site. These slopes are typical values and have been reported to reduce O_2_ in an alkaline medium catalyzed by macrocyclic complexes of Fe [33], mainly through the mechanism via four electrons to give water [21,22,34]. As expected, for the system with the highest covering (ML), a higher Tafel slope value was obtained according to the electrocatalysis results (Figure 5b,c). The value of −68 mV/dec is congruent with that reported in the literature for an ORR mechanism via two electrons to give peroxide [21,22].

## 3. Materials and Methods

All chemical precursors were reagent grade; tetrabutylammonium perchlorate (TBAP), 4-nitrobenzenediazonium tetrafluoroborate (NBTF), 2,2-diphenyl-1-picrylhydrazyl (DPPH), iron (II) phthalocyanine (FePc), sulfuric acid 95–97% (H_2_SO_4_), sodium hydroxide 99% (NaOH), and potassium hydroxide (via EMSURE^®^ (KOH) analysis) were purchased from Merck^®^ and used as received. Acetonitrile (ACN) and *N*,*N*-dimethylformamide (DMF) solvents were dried through conventional methods and distilled under nitrogen prior to use. Electrolyte solutions were prepared using a Britton–Robinson buffer solution (pH = 11). All solutions were purged with ultrapure nitrogen or oxygen for 30 min prior to electrochemical measurements, which were carried out using an Epsilon E2 BAS Potentiostat (Bio-Analytical Systems, West Lafayette, IN, USA) and a three-compartment electrochemical cell. For the nonaqueous electrolyte solution, an Ag/Ag^+^ reference electrode (0.01 M AgNO_3_ in dry ACN) was used, whereas for the aqueous solution a saturated calomel electrode and a platinum spiral-like auxiliary electrode was used. The working electrode was a gold slide annealed with a hydrogen flame. Prior to the heat treatment, the electrode was submerged in a “piranha” solution. Finally, the gold electrode was subjected to an electrochemical pretreatment, applying potential cycles between −0.4 and +1.1 V in H_2_SO_4_ (0.5 M) at 0.1 V/s scan rate until a stable and typical voltammogram was obtained.

The different relative covering degrees of the grafted layers; monolayer (*mL*-ArNO_2_) and two kind of more massive surface coverage (intermediate, *i*-ArNO_2_, and multilayer, *ML*-ArNO_2_) were obtained starting from 1 mM NBTF, with 0.1 M TBAP as the supporting electrolyte in dry ACN. For the *mL*-ArNO_2_ and *i*-ArNO_2_ covering degrees, two potential cycles ranging from 0.49 to −0.31 V (*v* = 0.05 Vs^−1^) were applied following a procedure in the literature [30]; additionally, the intermediate film (*i*) was obtained without DPPH and the monolayer film (*mL*) was obtained when 2 mM of DPPH was added to the electrolyte solution. The most massive surface coverage, multilayer *ML*-ArNO_2_, was obtained when a fixed potential of −0.1 V was applied for 60 s [39]. Later on, the modified electrodes were sonicated in ACN for 1 min. Then, during all cases, the end groups of grafted Ar–NO_2_ were reduced to Ar–NH_2_ by cyclic voltammetry between the 0.0 and −1.1 V, using the Britton–Robinson buffer solution, pH 11 [25,33]. With the multilayer system (*ML*-ArNO_2_/*ML*-ArNH_2_), a chemical reduction was performed with a 1 mM SnCl_2_ in 0.1 M HCl solution at 80 °C for 2 h.

Electrochemical impedance spectroscopy (EIS) studies were carried out at room temperature using a PGSTAT302N Metrohm Autolab (NL) potentiostat with a FRA32M module. The EIS measurements were executed at an open circuit potential (OCP) with a 5 mV (peak-to-peak) AC voltage in a frequency ranging from 0.1 MHz to 100 mHz in 2.5 mM [Fe(CN)_6_]^3−^/^4−^, each in 0.5 M KCl. After modification, the electrodes were incubated in a 0.1 g/L (1.76 × 10^−4^ M) FePc solution in dry DMF for 15 h. The electrode was then washed with DMF. The electrocatalysis of ORR was carried out by cyclic (CV) and linear voltammetry (LV). The CV was registered at 50 mV s^−1^ in 0.1 M NaOH saturated O_2_, and LV at 1 mV s^−1^ in 0.1 M NaOH in saturated O_2_.

The study of the surface morphology of modified electrodes was performed by scanning tunneling microscopy (STM) in an Agilent/Keysight 9500 Microscope (Santa Rosa, CA, USA), equipped with a STM Atomic Scanner (0.1 nA/V sensitivity) in constant-current mode with a typical bias voltage between 0.2 and 1.0 V and a set point current of 0.05–1 nA. The STM tips used were Pt–Ir tips (N9801A) electrochemically etched, bought from Keysight Technologies, (Santa Rosa, CA, USA).

## 4. Conclusions

We prepared gold-modified electrodes by the electrografting of nitro-aryl films with effective control of the covering level. Grafted films with different coverings were obtained; monolayer, *mL*-ArNO_2_; intermediate, *i*-ArNO_2_; and multilayer, *ML*-ArNO_2_. The surface-bounded nitrophenyl groups were electrochemically reduced and characterized by cyclic voltammetry, electrochemical impedance spectroscopy, and scanning tunneling microscopy. The Ar–NH_2_ terminal function generated on the grafts acted as the fifth axial ligand for coordination of the iron (II) phthalocyanine (FePc) catalyst complex on the modified electrode surface. The electrocatalytic study showed that grafted configurations with the lowest covering levels provided the best conditions from an electronic and a structural point of view to optimize the interaction between the O_2_ molecule and FePc active sites, promoting the ORR via the four-electron mechanism. The intermediate covering, *i-*ArNH_2_/FePc, showed the best electrocatalytic performance and generated the highest current density and a shift to more positive potentials for onset ORR.

## Figures and Tables

**Table 1:** Surface covering calculated from the voltametric response of nitrophenyl groups for modified surfaces.

Systems	ArNO_2_ to ArNH_2_ Process ^1^	ArNO_2_ to ArNHOH Process ^1^	Total ^1^
*mL*-ArNH_2_	1.78 × 10^−10^	1.37 × 10^−11^	1.91 × 10^−10^
*i*-ArNH_2_	4.40 × 10^−10^	1.55 × 10^−10^	5.96 × 10^−10^
*ML*-ArNH_2_	4.89 × 10^−10^	1.73 × 10^−10^	6.62 × 10^−10^

^1^ Covering (mol cm^−2^).

**Table 2:** Circuit parameters ^1^ from impedance data (average solution resistance: 48.5 Ω).

Covering	R*_ct_*/kΩ		C*_dl_*/μFcm^−2^	
	ArNO_2_	ArNH_2_	ArNO_2_	ArNH_2_
*mL*-ArNH_2_	4.70	4.04	0.60	2.82
*i*-ArNH_2_	14.4	4.13	0.48	1.65
*ML*-ArNH_2_	21.9	7.68	0.47	0.75

^1^ From Randles’ model.

## Data Availability

Not applicable.

## Sample Availability

Samples of the compounds are not available from the authors.
